# Essential Factors in Denture Construction

**Published:** 1922-01

**Authors:** Norman S. Essig


### ESSENTIAL FACTORS IN DENTURE CONSTRUCTION

BY NORMAN S. ESSIG

For a great many years we have had prosthetists who have been capable of obtaining results which are satisfactory both from the standpoint of efficiency and the artistic. Then came the scientist who proposed to settle all these matters from an ultra-scientific standpoint, and as these things multiplied the rank and file of the dental profession became mystified, and their viewpoint so clouded that they knew not what to do. Many lost interest entirely because they seemed to feel that while there was something in all these mathematics, they could not understand it, or perhaps they had no taste for figures. We have but to look back a few years and see how everything in prosthetics yielded to the various bands of researchers, and here we must divide these cults or schools under their respective banners. We have always had searchers after the truth, we have always had men who adopted new and intricate ideas and paraphernalia for the sole purpose of giving the appearance of scientific research; we have also those who have been working under the guise of searchers for the truth, but in reality were after the great round dollar, brooking no interference or difference of opinion from others in their efforts to commercialize.

Those who are really searching for the truth may be divided into two camps, *viz.*, those who try to simplify and make use of nature in her different moods and tenses, and those whose tendency is to standardize, and make a mathematical problem rather than anything else; in other words, they are trying to measure the works of God Almighty with a yardstick, as someone has tersely put it.

Let us ask ourselves as prosthetists several questions: What do we want? What do we find? What can we use? Why do we use it? How may the profession profit by it?

We may satisfy ourselves as to the anatomy and physiology, the physics and all that pertains to the natural functioning of the human mandible and maxillæ, yet we must put such data away in our note-book among our mental accomplishments, and start our plan upon the foundations which the patient brings with him when he enters our office. We must take what we find with its peculiarities, its limitations, its advantages, and its disadvantages, and build upon that a denture with which the patient can do efficient work, and which will be artistic enough that the eye may not be offended. We must not, I may say, can not adhere to any school. We must substitute atmospheric adhesion for all the other forms of attachment which were present under normal conditions. We must adhere to the different steps in the technic of this work, because it is the only thing that we can use. We employ these different steps in different degrees in the mouths that present themselves to us, and the variance which presents itself in each case complicates our work. I believe that too little attention has been paid to the human articulator. We have been neglecting for a great many years a great opportunity to use this instrument, which is the only anatomical articulator in existence. I am sure the profession can be assisted, the work made easier, and results much better, by taking advantage of the natural data wherever possible.

If we can cause our patients to check up our work as we go along, if we can make use of this human articulator in acquiring the compensating curve, the question as to whether or not the human jaw is a lever of the first or second class, or conforms to an equilateral or isosceles triangle, will sink into insignificance immediately. If our denture is firm and has artistic qualities and the patient is able to forget its presence, a great many of the more scientific phases become of no value as such, except as a mental accomplishment, because they can not be pressed into service in making a denture.

If the bite waxes are armed with abrasive material after care and time have been bestowed upon the adjustment, and the patient instructed how to chew or make the movements of mastication, the planes we have made will be greatly modified and changed, and will, if the teeth be set up on these lines, give greater ease and comfort during the process of mastication. The patient in the case will record both the long and the short curves, and will place them in their proper positions, a thing we dentists could not do in any case.

It was Dr. Villain who first directed my attention to the fact that teeth were articulated upon an angle, the apex of which was somewhere in the forehead, and this triangle directed the long axis of each tooth, so that the stress of mastication came in the right direction. I found upon further investigation that the occlusal plane was governed and controlled by this triangle and the size and width of the mandible which turned inward to accommodate the arch in the maxillæ, which is generally smaller than that in the mandible.

I believe with Dr. Wilson that most of the devices or instruments in use today are scientific fiddles upon which one may play his pet tune, and the profession in general can play any tune it happens to know.

In my experience there is no cusp angle that can be made universal, nor do I believe that within reason one is any more efficient than another in the mastication of food. After measuring many natural bicuspids the average was found to be about 36, which has in my hands proved very satisfactory.

Too little stress has been laid upon the occlusal plane as evolved by the possibilities and limitations of the mandible. We should, when possible, idealize the curve of Spee, but the patient must modify the curve to allow ease in mastication, and this can not be done by an instrument.

The curve of Spee is a most valuable asset to the patient in many cases, because like the scimiter, this curve performs a slicing or cutting function no matter how the mouth is closed or the mandible moved, whereas a straight line will crush like the straight-bladed sword, often making a clicking noise. This curve makes for efficiency, and often where there are other limitations the denture can be made efficient by this means when denied a variety of movements common to others.

In summarizing his paper, Dr. Wilson has sounded the all-important note in our work as prosthetists, when he says that the essential factor in dental construction is mental rather than instrumental. This must ever be. because of the data he has given us today, because of the great variation in measurements, because of the variation from the ideal in anatomy and physics of prosthetics, and because of the artistic phase which is as important as any of the other steps in denture construction.

The result of research thus far seems to have complicated dental prosthetics, and I personally feel that the same efforts expended in the simplification of our work, the following of nature's plan, and the close observance of this side of the question would have been productive of much better results.

Our attitude toward mechanical devices should be a broad one, yet up to the present day all that we can ask of any such instrument is that it will maintain the relation of the maxillæ and mandible during the absence of the patient and while certain steps in the construction of any denture shall be carried to completion, and until the return of the patient for adjustment in the mouth. There can be no such thing as standardization in denture construction.—*Dental Cosmos,* page 1311, December, 1921.

